# Upregulation of Aquaporin 1 Mediates Increased Migration and Proliferation in Pulmonary Vascular Cells From the Rat SU5416/Hypoxia Model of Pulmonary Hypertension

**DOI:** 10.3389/fphys.2021.763444

**Published:** 2021-12-17

**Authors:** Xin Yun, Nicolas M. Philip, Haiyang Jiang, Zion Smith, John C. Huetsch, Mahendra Damarla, Karthik Suresh, Larissa A. Shimoda

**Affiliations:** Division of Pulmonary and Critical Care Medicine, Johns Hopkins School of Medicine, Baltimore, MD, United States

**Keywords:** smooth muscle cells, pulmonary arterial hypertension, microvascular endothelial cells, lung, remodeling

## Abstract

Pulmonary arterial hypertension (PAH) is a progressive disorder characterized by exuberant vascular remodeling leading to elevated pulmonary arterial pressure, maladaptive right ventricular remodeling, and eventual death. The factors controlling pulmonary arterial smooth muscle cell (PASMC) and endothelial cell hyperplasia and migration, hallmark features of the vascular remodeling observed in PAH, remain poorly understood. We previously demonstrated that hypoxia upregulates the expression of aquaporin 1 (AQP1), a water channel, in PASMCs, and that this upregulation was required for hypoxia-induced migration and proliferation. However, whether the same is true in a model of severe PAH and in pulmonary microvascular endothelial cells (MVECs) is unknown. In this study, we used the SU5416 plus hypoxia (SuHx) rat model of severe pulmonary hypertension, which mimics many of the features of human PAH, to determine whether AQP1 levels were altered in PASMCs and MVECs and contributed to a hyperproliferative/hypermigratory phenotype. Rats received a single injection of SU5416 (20 mg/kg) and then were placed in 10% O_2_ for 3 weeks, followed by a return to normoxic conditions for an additional 2 weeks. We found that AQP1 protein levels were increased in both PASMCs and MVECs from SuHx rats, even in the absence of sustained hypoxic exposure, and that in MVECs, the increase in protein expression was associated with upregulation of AQP1 mRNA levels. Silencing of AQP1 had no significant effect on PASMCs from control animals but normalized enhanced migration and proliferation observed in cells from SuHx rats. Loss of AQP1 also reduced migration and proliferation in MVECs from SuHx rats. Finally, augmenting AQP1 levels in MVECs from control rats using forced expression was sufficient to increase migration and proliferation. These results demonstrate a key role for enhanced AQP1 expression in mediating abnormal migration and proliferation in pulmonary vascular cells from a rodent model that reflects many of the features of human PAH.

## Introduction

Pulmonary hypertension (PH) is a devastating, progressive condition of varying etiology leading to eventual right heart failure. Clinically diagnosed as a mean pulmonary arterial pressure >20 mmHg, PH can be divided into five categories based on clinical manifestations, hemodynamics, inciting cause, and response to therapies (Simonneau et al., 2019). PH associated with hypoxia (Group 3) is common in individuals with chronic lung disease or non-adapted individuals who live at high altitudes, whereas the most severe form of PH is disease predominating in the pre-capillary circulation, classified as pulmonary arterial hypertension (PAH; Group 1). Mortality with PAH is unacceptably high, with 5-year survival at approximately 50% (Benza et al., 2012). Vascular remodeling is a prominent feature of all forms of PH, but is especially robust in PAH, with thickening of all layers of the vascular wall and development of occlusive lesions that obstruct blood flow in the small arteries (Tuder, 2009; Hemnes and Humbert, 2017; Humbert et al., 2019). While vascular remodeling is a known contributor to the elevation in pulmonary vascular resistance and pressure during PH, no current therapies are available that directly target remodeling, partly because the cellular mechanisms governing this process remain poorly understood.

In previous work, we demonstrated that hypoxia upregulates the protein expression of the water channel, aquaporin 1 (AQP1), in pulmonary arterial smooth muscle cells (PASMCs) and identified a role for this protein in mediating proliferation and migration in response to hypoxia (Leggett et al., 2012). Following this initial study, other labs confirmed the role of AQP1 in governing PASMC growth during hypoxia and showed that targeting AQP1 *in vivo* reduced the development of hypoxia-induced PH in rodents (Schuoler et al., 2017; Liu et al., 2019). While these studies appear to cement a role for AQP1 in hypoxia-induced PH, the role of AQP1 in pulmonary vascular cell growth and migration in PAH, where sustained hypoxia is not an inciting or complicating factor, is unknown. At present, no preclinical model perfectly recreates human PAH; however, the model produced by injecting rats with a vascular endothelial growth factor receptor inhibitor (SU5416) combined with hypoxic exposure followed by a return to normoxic conditions for several weeks recapitulates several important features of human PAH, including severe elevations in pulmonary arterial pressure and robust vascular remodeling including vaso-occlusive lesions (Taraseviciene-Stewart et al., 2001; Abe et al., 2010; Huetsch et al., 2016). We have previously shown that PASMCs and pulmonary microvascular endothelial cells (MVECs) isolated from this model exhibit several abnormalities, including enhanced migratory and proliferative capacity (Huetsch et al., 2016; Suresh et al., 2018, 2019). The current study tested the hypothesis that AQP1 is upregulated in PASMCs and MVECs from the SuHx rat model and contributes to the abnormal growth and migration observed in these cells.

## Materials and Methods

### Ethical Approval

All procedures and protocols in this study were conducted in accordance with NIH guidelines for the proper care and use of animals in research and were approved by the Johns Hopkins University Animal Care and Use Committee.

### Sugen/Hypoxia Model

To induce PH, adult male Wistar rats (150–200 g) received a subcutaneous injection of SU5416 (20 mg/kg) prepared in a carboxymethylcellulose (CMC)-containing diluent as described previously (Taraseviciene-Stewart et al., 2001; Huetsch et al., 2016) and were exposed to 10% O_2_ (hypoxia) for 3 weeks. During this time, the rats were exposed to normoxic conditions for <5 min twice a week to change cages and replenish food and water. At the end of 3 weeks, rats were returned to room air (normoxia) for an additional 2 weeks. Control rats were injected with vehicle and maintained in room air for 5 weeks. All animals were kept in the same room and exposed to the same light-dark cycle and ambient temperature and were housed in standard rat cages (three rats/cage) with free access to food and water. At the end of exposures, rats were anesthetized (Ketamine, 75 mg/kg; Xylazine, 7.5 mg/kg). Depth of anesthesia was confirmed *via* paw pinch prior to measurement of the right ventricular systolic pressure (RVSP) *via* transdiaphragmatic right heart puncture with a heparinized 23 gauge needle attached to a pressure transducer as previously described (Huetsch et al., 2016; Suresh et al., 2018). Animals were euthanized *via* exsanguination after hemodynamic measurements and the heart and lungs removed and transferred to a dissecting dish filled with cold N-[2-hydroxyethyl]piperazine-N′-[2-ethanesulfonic acid] HEPES-buffered salt solution (HBSS) containing (in mmol/L): 130 NaCl, 5 KCl, 1.2 MgCl_2_, 1.5 CaCl_2_, 10 HEPES, and 10 glucose, with pH adjusted to 7.2 with 5 mol/L NaOH. Under a microscope, the atria and large conduit vessels were removed, and the right ventricular (RV) wall was carefully separated from the left ventricle and the septum (LV + S). Both portions were blotted dry and weighed.

### Isolation of Smooth Muscle Cells

Pulmonary arterial smooth muscle cells were isolated as described previously (Shimoda et al., 1998). Lungs were placed in cold HBSS and intrapulmonary arteries (PAs; 200–600 μm outer diameter) were isolated under a dissecting microscope and cleaned of adventitia and endothelium. The arteries were allowed to recover for at least 30 min on ice before being transferred to reduced-Ca^2+^ (20 μM CaCl_2_) HBSS at room temperature for at least 20 min. The tissue was digested for 20–25 min at 37°C in reduced-Ca^2+^ HBSS containing collagenase (type I; 1,750 U/ml), papain (9.5 U/ml), bovine serum albumin (2 mg/ml) and dithiothreitol (1 mM). PASMCs were obtained by gentle trituration in Ca^2+^-free HBSS using a modified P1000 pipette tip and cultured in Smooth Muscle Cell Medium supplemented with SmGM™-2 bullet kit (Lonza). PASMC phenotype and purity were validated by assessing the presence of smooth muscle-specific actin (SMA) using immunofluorescence in cells incubated with anti-SMA (1:1,000, Sigma) primary antibody and counterstained with DAPI. PASMCs were used at passages 0–3.

### Isolation of Endothelial Cells

Microvascular endothelial cells were isolated, grown to confluence, and phenotyped as described previously (Suresh et al., 2018, 2019). Peripheral strips of rat lung were dissected and digested in reduced Ca^2+^ HBSS containing collagenase type 1 (357 U/ml for 30 min). Following digestion, cells were incubated for 30 min with CD31-conjugated dynabeads (1:40, #11155D, Invitrogen) and then CD31+ cells magnetically selected, plated in DMEM media containing non-essential amino acids, endothelial growth factor supplement (Millipore), and 20% fetal bovine serum (FBS; Hyclone) and 1% penicillin/streptomycin as described previously (Suresh et al., 2018, 2019) and grown to confluence. Cells were then subjected to a second selection using *Griffonia simplicifolia*-conjugated beads (1:2,000, #B-1105, Vector; or 1:40, #11047, Invitrogen). Following dual selection, cells were used or frozen at passage 3. All experiments were performed on cells at passages 3–4. MVECs were routinely phenotyped for lack or presence of smooth muscle (SMA; 1:200, FITC-conjugated smooth muscle-specific α-actin; # F3777, Sigma) and EC (von Willebrand factor, vWF; 1:100, #SARTW-1G, Affinity Biologicals and Donkey anti-sheep Alexa Fluor, 1:100, #A21436, Invitrogen) markers, respectively. Cells were counterstained with DAPI (1 μg/ml in PBS, #62247, ThermoFisher Scientific) to visualize nuclei.

### Real-Time RT-PCR

Total RNA was extracted from PAs, PASMCs, and MVECs *via* the RNeasy Plus Mini kit (Qiagen). Reverse transcription was performed using 500 ng of RNA and the iScript cDNA synthesis kit (Bio-Rad). Real-time PCR was performed using 1,000 ng of cDNA with QuantiTect SYBR Green PCR Master Mix (Qiagen) in an iCycler IQ real-time PCR detection system (Bio-Rad). The specific primer pairs for real-time PCR are listed in Table 1. Confirmation of correct PCR products was achieved by observation of a single peak in the melt curve and visualization of a single band of the correct size when products were run on an agarose gel. Finally, bands were excised and sequenced. Relative amounts of each gene were calculated using the Pfaffl (2001) method, and data are expressed as a power ratio of the gene of interest to the housekeeping gene (Cyclophilin B; CpB) within a sample using the efficiency for each gene calculated from efficiency curves run on the same plate as samples. For each experiment, the values were normalized to the value of the control sample.

**Table 1:** Real-time PCR primers.

Gene name	Accession Number	Forward	Reverse	Product size (kB)
AQP1	NM 012778	5'-CCGAGACTTAGGTGGCTCAG-3'	5'-TTGATCCCACAGCCAGTGTA-3'	118
Cyclophilin B	NM 022536	5'-GGACGAGTGACCTTTGGACT-3'	5'-TGACACGATGGAACTTGCTG-3'	93

### Forced Expression of AQP1

An adenoviral construct containing wild-type AQP1 with an N-terminal hemagglutinin (HA)-tag (AdAQP1) was created as described previously (Lai et al., 2014). The same adenoviral construct containing GFP (AdGFP) was used as a control for infection. MVECs were infected with 100 ifu/cell of virus in low serum (1% FBS) media for 6 h after which media was replaced and cells allowed to incubate an additional 24 h before being used in experiments.

### siRNA Transfection

Depletion of endogenous AQP1 was achieved using siRNA targeting AQP1 and non-targeting siRNA (control) obtained as a “smart pool” (Dharmacon) as previously described (Leggett et al., 2012). PASMCs and MVECs were incubated with 100 nM of siRNA for 6 h in serum- and antibiotic-free media, after which serum was added to media for a total concentration of 0.3% (PASMCs) or 5% (MVECs) FBS. Cells were incubated under these conditions for 24 h, and then media was replaced, and cells were incubated for an additional 24 h prior to experiments.

### Western Blotting

Total protein was extracted from PAs, PASMCs, and MVECs in ice-cold T-PER buffer containing protease inhibitors (Roche Diagnostics). Quantification of proteins was performed using BCA protein assay (Pierce) and 10 μg (PAs and PASMCs) or 25 μg (MVECs) total protein per lane was separated by electrophoresis through a 12% SDS-PAGE gel and transferred onto polyvinylidene difluoride membranes. Membranes were incubated in 5% non-fat dry milk in Tris-buffered saline containing 0.2% Tween 20 to block nonspecific binding sites before being probed with primary antibody against AQP1 (1:1,000; # AQP11-A, Alpha Diagnostic Intl. Inc.) or HA (1:500; #sc-7392, Santa Cruz Biotechnology. Inc). Bound antibody was probed with horseradish peroxidase-conjugated anti-rabbit or anti-mouse secondary antibody (1:10,000; #5220-0336 and 5220-0341; KPL) and detected by enhanced chemiluminescence. Membranes were then stripped and re-probed for β-tubulin (1:10,000, #T7816, Sigma) as a housekeeping protein. Protein levels were quantified by densitometry using ImageJ.

### Cell Migration

Fifty-thousand cells in low FBS media (0.3% for PASMCs or 5% for MVECs) were added to the top chamber of a polycarbonate transwell insert (8 μm pores) inserted into 6-well plates. Cells were allowed to migrate in low serum media for 16 h (MVECs) or 24 h (PASMCs), after which the cells were fixed in 95% ethanol for 10 min, stained with Brilliant Blue (Pierce) for 5 min, and visualized *via* a microscope-mounted camera. For each filter, five fields were randomly chosen and imaged with Q-capture software (total cells). Unmigrated cells were then scraped off from the top of the filter, and the bottom layer was re-imaged (migrated cells). Migration rate was calculated as the percent of cells remaining on the filter after scraping normalized to the total amount of cells.

### Cell Proliferation Assay

Five-thousand cells were seeded into low FBS media (0.3% for PASMCs or 5% for MVECs) in wells of a 96-well plate in triplicate for every sample. After incubating for 2–3 h to allow cells to adhere, BrdU (Amersham Biosciences) was added to each well for 16 h (MVECs) or 24 h (PASMCs), after which the media was removed, and cells were fixed. Proliferation was estimated from the detection of peroxidase- labeled anti-BrdU in newly synthesized cells. The developed color was measured at 450 nm in a microtitre plate spectrophotometer. Proliferation rate is shown as the percent of the absorbance values normalized to the control cells on the same plate, with control values set to 100%.

### *In vitro* Exposure to Hypoxia

Microvascular endothelial cells were grown to 60% confluence in tissue culture plates and placed in low serum media for 4–6 h before beginning experiments. Cells were treated with vehicle (1:100 DMSO) or TC-S 7009 (10 μM) an inhibitor of hypoxia-inducible factor 2 (HIF-2) immediately prior to placing the culture plates in a modular chamber (Billups-Rothberg) gassed with 4% O_2_; 5% CO_2_ as previously described (Leggett et al., 2012). The chamber containing cells was placed in a room air incubator maintained at 37°C and 5% CO_2_. Correct oxygen levels inside the hypoxic chamber were confirmed using a hand-held oxygen monitor (model 5,577; Hudson RCI). Culture plates containing non-hypoxic control cells were placed in the same incubator on the shelf next to the modular hypoxia incubator.

### Electric Cell-Substrate Impedance Sensing

Endothelial cell barrier function was measured using electric cell-substrate impedance sensing (ECIS; ZTheta system, Applied Biophysics) as previously described (Suresh et al., 2015). MVECs (25,000 cells per well) were plated on gold electrodes in 0.5-ml electrode wells (Applied BioPhysics) and grown to confluence (48 h) in media containing 5% FCS. Baseline resistance for all wells was measured for an additional hour and an average resistance over this time was calculated.

### Statistical Analysis

Data are expressed as means ± SD, where “*n*” refers to the sample size. Since all experimental runs were performed on arteries/cells from different animals, “*n*” also refers to the number of animals. Statistical comparisons were performed using parametric or non-parametric tests as appropriate. Differences between groups were assessed by Student’s *t*-test, one-sample *t*-test, Mann-Whitney Rank Sum test, or two-way ANOVA with Holm-Sidak post-test. The specific statistical tests used in each experiment are detailed in the Figure legends. Differences were considered to be significant when *p* < 0.05.

## Results

### Model Validation

**Figure 1:**
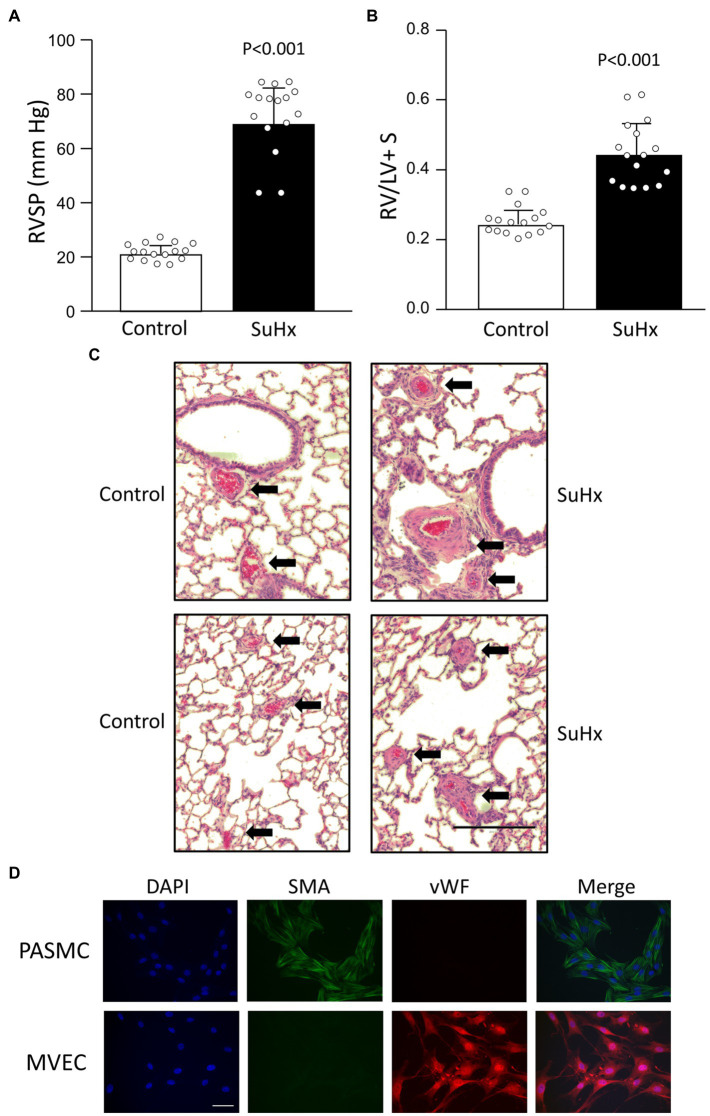
Validation of model. Bar graphs showing mean ± SD values for **(A)** right ventricular systolic pressure (RVSP) and **(B)** right ventricle (RV) to left ventricle plus septum (LV + S) weight ratio in control and SuHx rats. Each dot represents a single animal. Value of *p* determined by *t*-test. **(C)** Representative histology images (H&E staining) illustrating vascular remodeling in SuHx rats. Dark red blood cells are clearly evident within vessels. Arrows point to arteries (top) and small vessels (bottom); bar indicates 200 μm. **(D)** Representative images showing immunofluorescence for DAPI (nuclei), smooth muscle-specific α-actin (SMA), von Willebrand factor (vWF), and the merged image for pulmonary arterial smooth muscle cells (PASMCs) and MVECs isolated from control rats. Scale bar indicates 50 μm. Results representative of those obtained in at least 10 isolates.

At the end of the 5 weeks exposure, SuHx animals weighed less than controls (Table 2). As expected, exposure to the SuHx protocol resulted in elevated RVSP (Figure 1A) and increased right ventricular mass, reflected in the higher RV/LV + S weight ratio (Figure 1B). While PAs in lungs from control animals are thin-walled structures, an inspection of lung sections confirmed substantial thickening of the wall of PAs in lungs from SuHx rats (Figure 1C, top). Further examination of small diameter (<100 μm) vessels revealed near occlusion of the vascular lumen in many vessels (Figure 1C, bottom). These results are indicative of the development of severe PH and marked vascular remodeling in these animals. Isolation of PASMCs and MVECs was confirmed by immunofluorescence staining. PASMCs exhibited robust staining for SMA, as seen in Figure 1D. In contrast, MVECs isolated from control rats lacked SMA expression and instead exhibited staining for vWF, indicative of normal EC function/phenotype.

**Table 2:** Hemodynamic and physiological measurements in Control and SuHx rats.

Condition	Weight (g)	RVSP (mmHg)	RV/LV + S	Heart Rate (bpm)
Control (*n* = 16)	398.5 ± 21.4	21.1 ± 3.1	0.243 ± 0.04	341.3 ± 55.1
SuHx (*n* = 16)	309.9 ± 42.2^*^	68.9 ± 13.3^*^	0.442 ± 0.09^*^	316.5 ± 41.3

*RVSP, right ventricular systolic pressure; RV, right ventricle; LV + S, left ventricle plus septum; bmp, beats per minute*.

* *p < 0.001*.

### AQP1 Expression in Pulmonary Cells From SuHx Model

**Figure 2:**
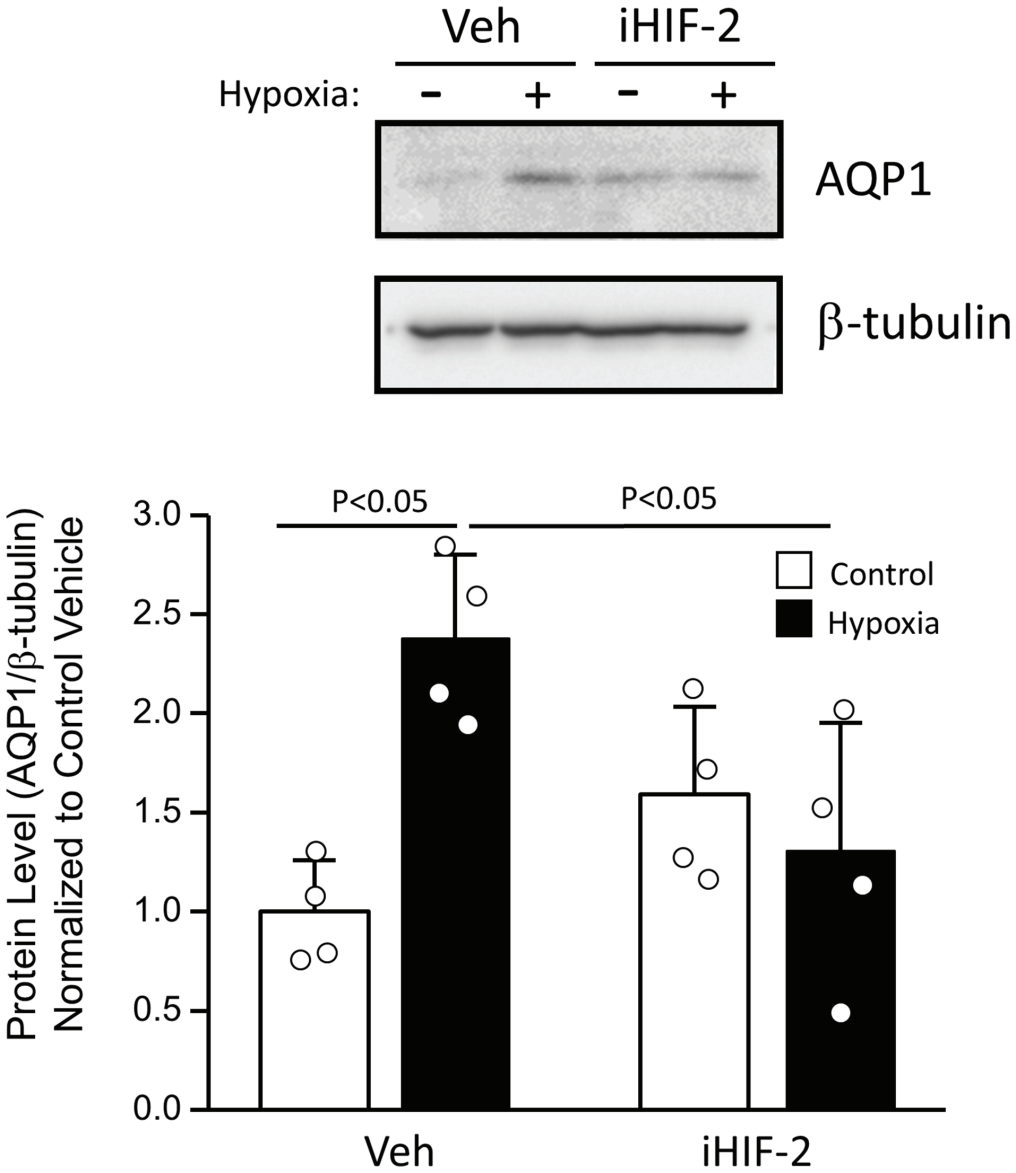
Effect of exposure to *in vitro* hypoxia on aquaporin 1 (AQP1) protein levels in MVECs. Representative immunoblot showing AQP1 and β-tubulin protein expression in MVECs exposed to 4% O_2_ for 48 h. Cells were treated with vehicle (Veh; 1:1,000 DMSO) or TC-S 7009 (10 μM), a hypoxia-inducible factor-2 (HIF-2) inhibitor (iHIF-2) prior to hypoxic challenge. Bar graphs show mean ± SD values for the ratio of AQP1 to β-tubulin protein levels, normalized to Control + Veh group. Value of *p* determined from two-way ANOVA with Holm-Sidak post-test. Each dot represents samples from different animals.

**Figure 3:**
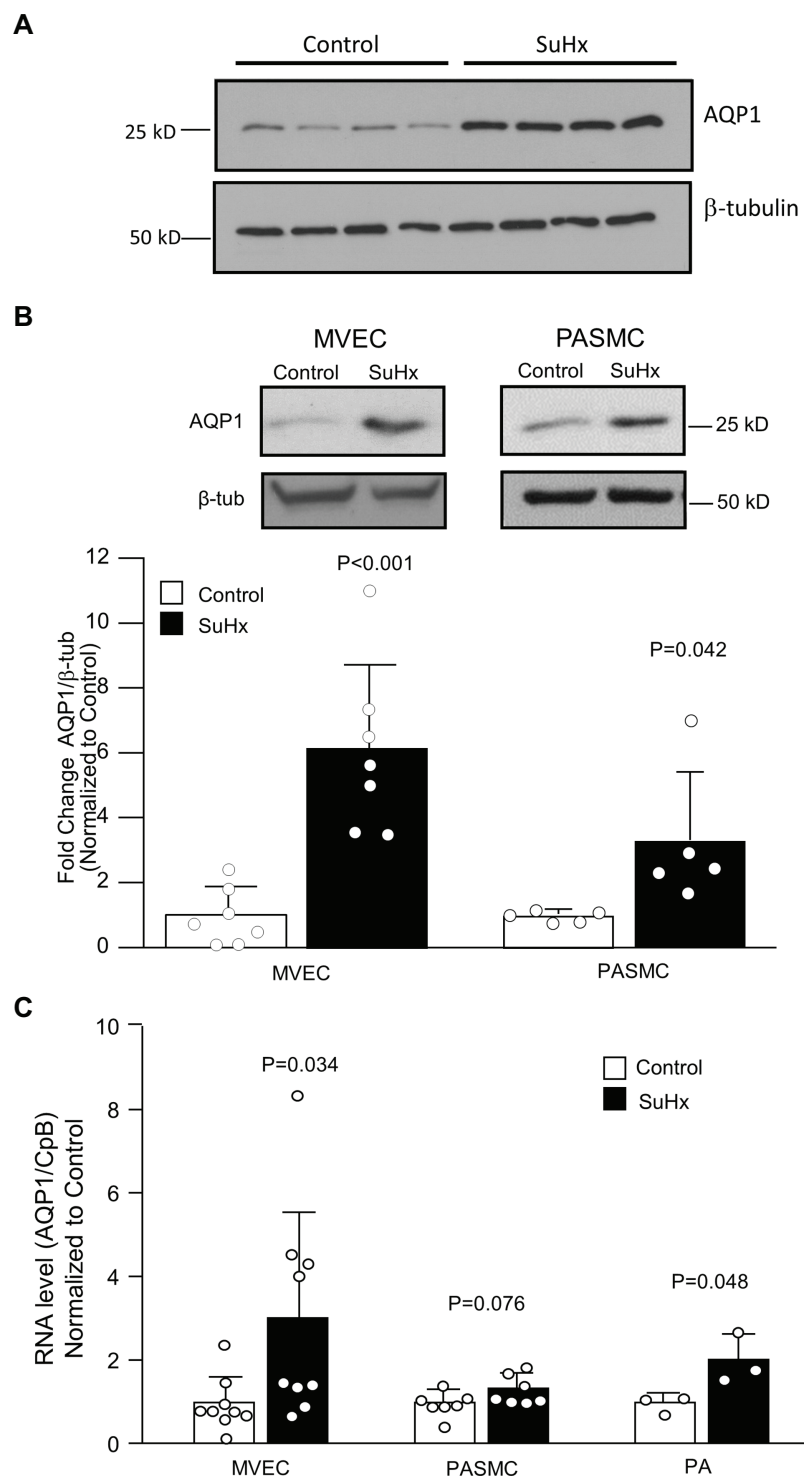
Pulmonary vascular AQP1 expression. **(A)** Immunoblot image showing AQP1 protein levels in pulmonary arteries (PA) from control and SuHx rats. **(B)** Representative images and bar graphs (mean ± SD values) for AQP1 protein levels in microvascular endothelial cell (MVEC) and PASMCs isolated from control and SuHx rats. **(C)** Bar graphs (mean ± SD values) for mRNA levels of AQP1 relative to cyclophilin B (CpB; housekeeping) and normalized to values in Control rat samples. For all graphs, each dot represents samples from different animals and values of *p* determined by *t*-test.

We previously demonstrated upregulated expression of AQP1 in PASMCs from chronically hypoxic rats and in PASMCs exposed to hypoxia *in vitro* (Leggett et al., 2012). To determine whether hypoxia could also increase AQP1 expression in MVECs, we exposed cells from control rats to 4% hypoxia for 48 h *in vitro*. Compared to MVECs kept in non-hypoxic conditions, MVECs exposed to hypoxia exhibited a modest increase in AQP1 protein levels (Figure 2). Since the AQP1 promoter had previously been demonstrated to contain a binding site for the oxygen-sensitive transcription factor, hypoxia-inducible factor (HIF; Abreu-Rodriguez et al., 2011; Tanaka et al., 2011), we also tested whether the upregulation of AQP1 by hypoxia in MVECs was HIF-dependent. Incubation with an inhibitor to HIF-2, the family member primarily expressed in endothelium, had no significant effect on AQP1 levels under non-hypoxic conditions but prevented the upregulation of AQP1 in MVECs exposed to hypoxia. We next determined whether AQP1 was upregulated in the vasculature of SuHx rats, we initially performed immunoblot analysis on whole PAs. Resistance level PAs from SuHx rats exhibited increased AQP1 protein levels compared to arteries from control rats (Figure 3A). We next assessed AQP1 expression in PASMCs and MVECs isolated from these animals. In both PASMCs and MVECs, AQP1 protein levels were significantly higher in cells from SuHx rats than controls (Figures 3B,C), although AQP1 expression in control MVECs was quite low and required loading more than double the amount of protein (25 μg) as loaded from PASMCs (10 μg) to see comparable levels of basal expression. Even in this case, AQP1 protein was nearly undetectable in several MVEC samples. In contrast to the protein expression findings, MVECs exhibited substantial AQP1 mRNA at baseline, with *C*_t_ values for AQP1 in control MVECs (mean *C*_t_ = 24.26) similar to values measured for AQP1 in control PASMCs (*C*_t_ = 22.9) in the same experiment. RT-PCR also revealed an increase in AQP1 mRNA levels in MVECs and PAs from normoxic and SuHx rats (Figure 3C), whereas AQP1 mRNA levels in PASMCs from control and SuHx rats were not significantly different.

### Migration and Proliferation in PASMCs and MVECs

**Figure 4:**
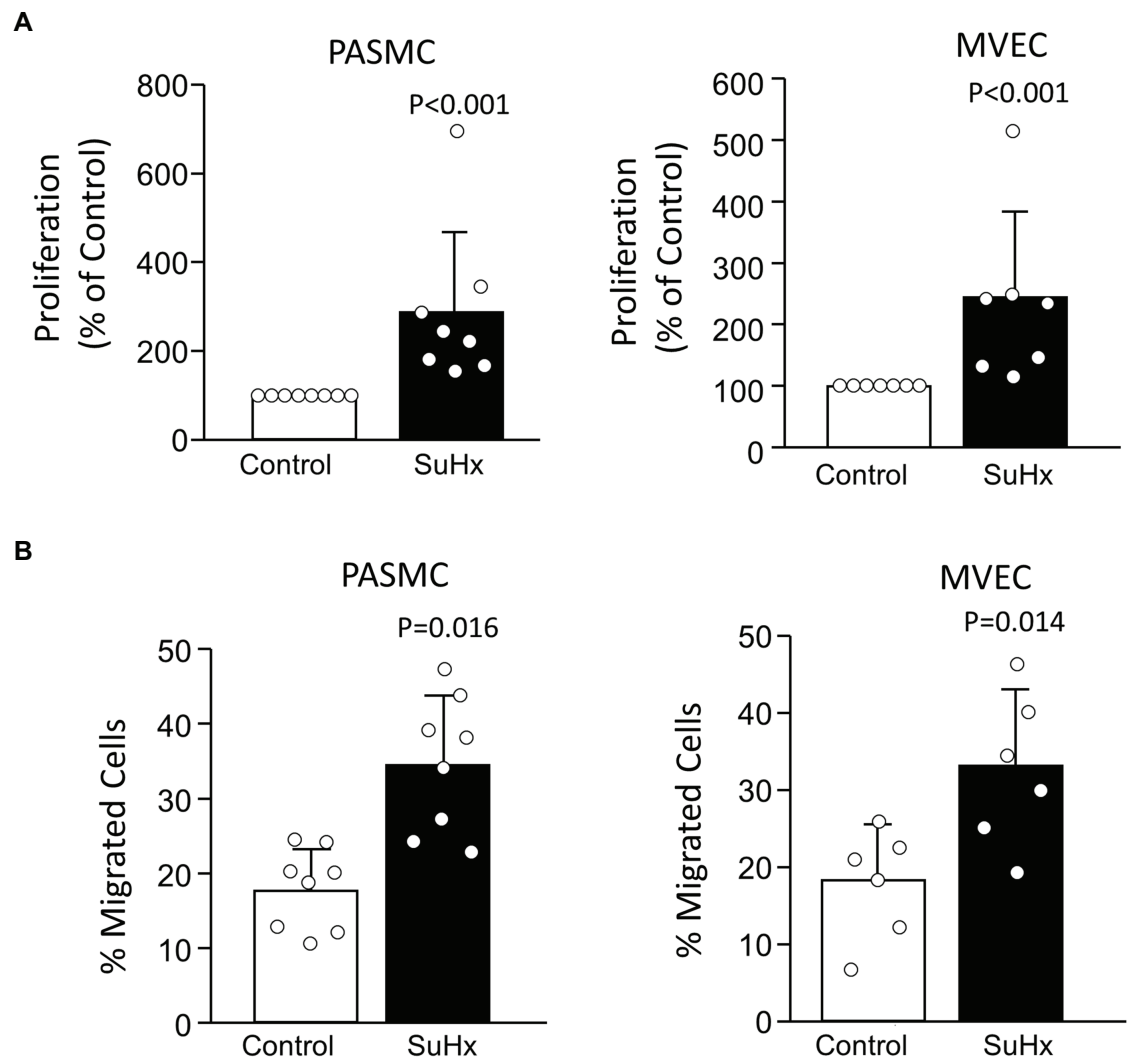
Proliferation and migration in cells from Control and SuHx rats. **(A)** Bar graphs (mean ± SD values) for proliferation, measured by BrdU incorporation normalized to Control value, in PASMC and MVEC isolated from control and SuHx rats. Values of *p* were determined from Mann-Whitney Rank Sum test. **(B)** Bar graphs (mean ± SD values) for migrated cells (% of total) were assessed by transwell assay using PASMCs and MVECs from control and SuHx rats. Values of *p* determined by *t*-test. For all graphs, each dot represents samples from different animals.

**Figure 5:**
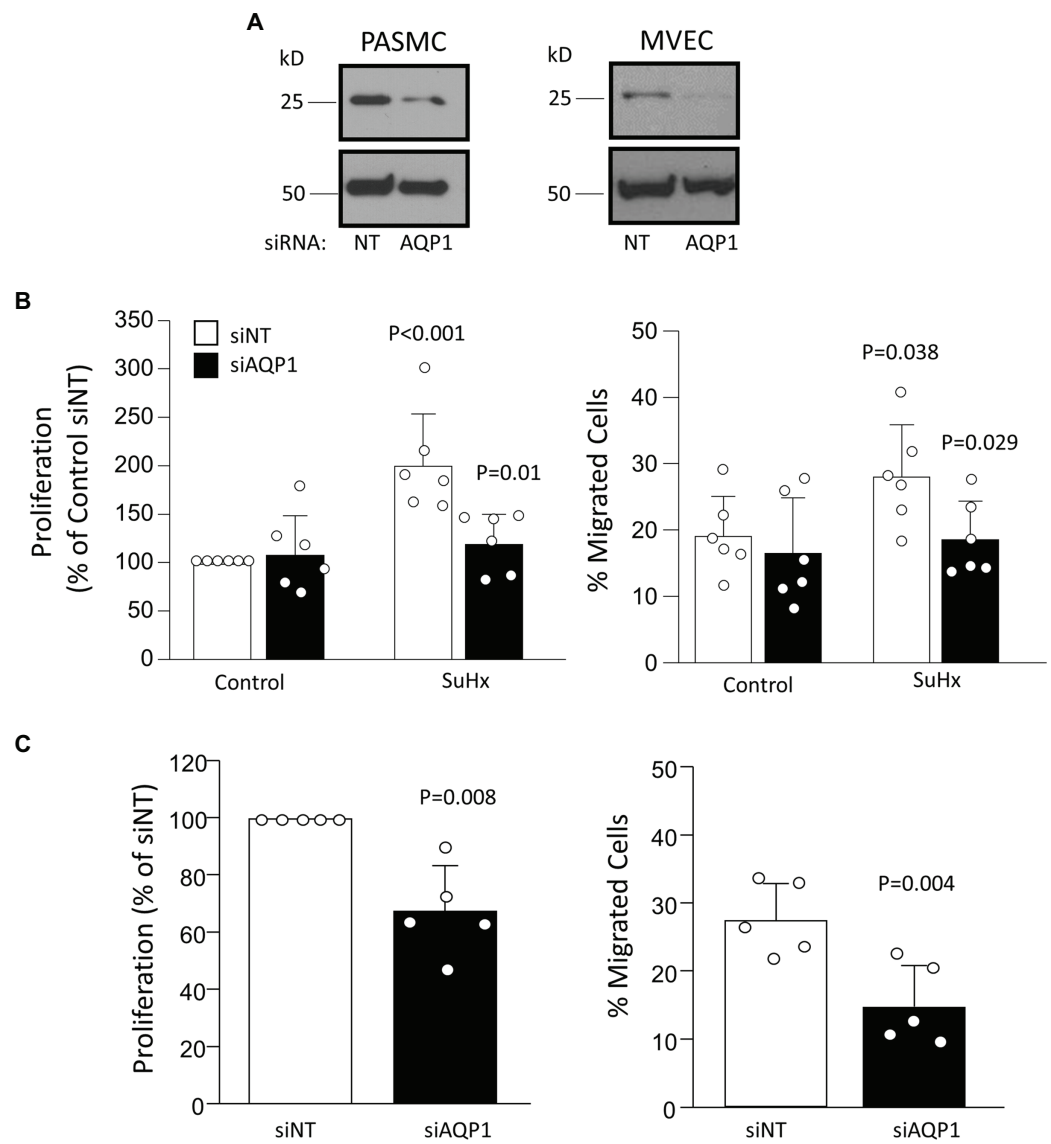
Effect of depleting AQP1 on proliferation and migration. **(A)** Representative images demonstrating level of protein knockdown achieved using siRNA directed against AQP1 (siAQP1) compared to non-targeting siRNA (siNT). Result representative of at least five experiments for each cell type performed on cells from different animals. **(B)** Bar graphs (mean ± SD values) for proliferation, measured by BrdU incorporation and normalized to control, and migration, measured *via* transwell assay, in PASMCs isolated from control and SuHx rats. Values of *p* for proliferation and migration in PASMCs were determined from two-way ANOVA with Holm-Sidak post test to determine differences between specific groups. **(C)** Bar graphs (mean ± SD values) for proliferation and migrated cells in MVEC from SuHx rats. Values of *p* determined by *t*-test (migration) or one-sample *t*-test (proliferation) against a mean of 100. For all graphs, each dot represents samples from different animals.

Compared to cells from control rats, PASMCs and MVECs from SuHx rats exhibited greater proliferative (Figure 4A) and migratory potential (Figure 4B), as expected based on results reported in our previous studies (Huetsch et al., 2016; Suresh et al., 2018). Using siRNA approaches, we were able to achieve a substantial reduction in AQP1 protein levels in both PASMCs and MVECs from SuHx rats (Figure 5A). Compared to cells transfected with non-targeting siRNA (siNT), depleting AQP1 reduced proliferation and migration in SuHx PASMCs (Figure 5B) but had no effect on migration or proliferation in control PASMCs. Since baseline levels of AQP1 protein were very low and often undetectable in control MVEC, we only performed depletion experiments in cells from SuHx rats. In SuHx MVECs, silencing AQP1 reduced proliferation by 33% and migration to ~15% (Figure 5C), very close to levels measured in MVECs from control rats as seen in Figure 4.

### Effect of Augmenting AQP1 on MVECs Migration and Proliferation

**Figure 6:**
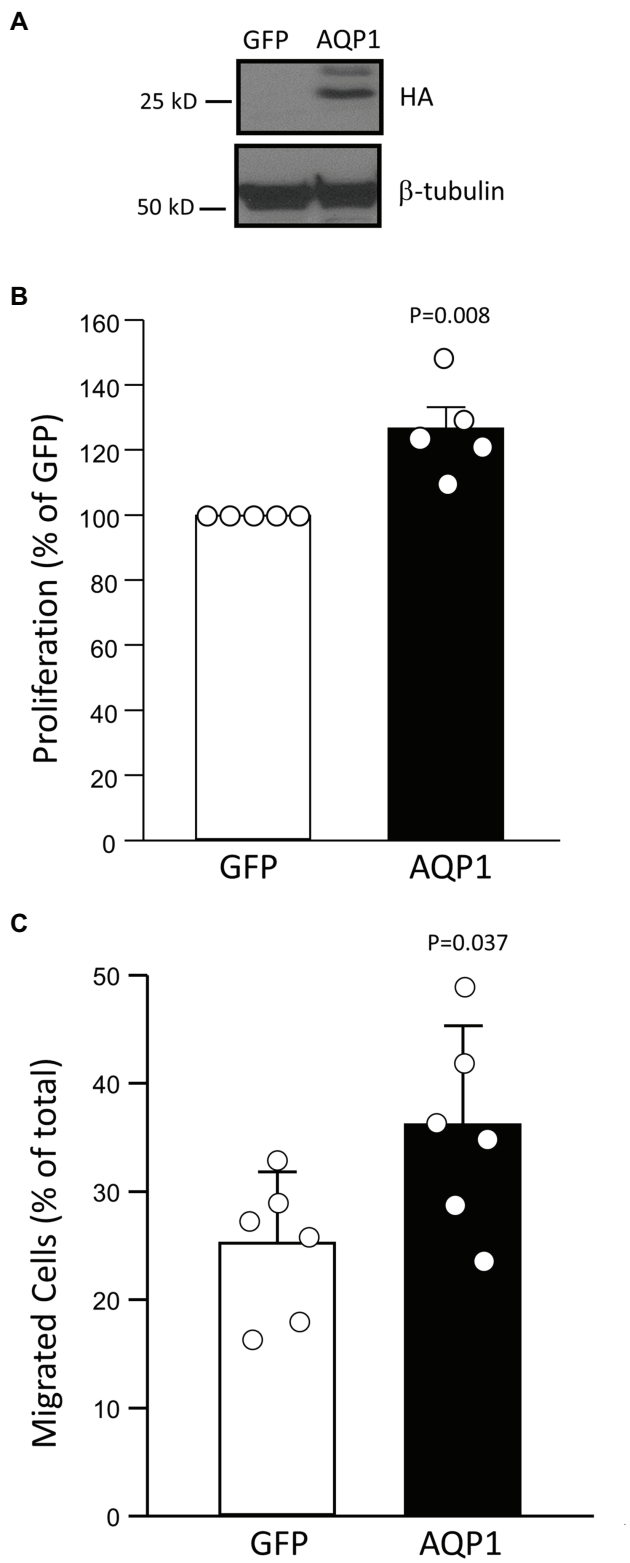
Effect of increasing AQP1 on proliferation and migration. **(A)** Representative images demonstrate a level of HA-tagged AQP1 protein expressed following infection with an adenovirus containing GFP or AQP1 compared to β-tubulin (loading control). **(B)** Bar graph (mean ± SD values) for proliferation, measured by BrdU incorporation and normalized to control, in pulmonary MVECs isolated from control rats and infected with GFP- or AQP1-containing virus. Value of *p* determined from Mann Whitney Rank Sum Test. **(C)** Bar graph (mean ± SD values) for migrated cells in MVECs from control rats infected with virus-containing GFP or AQP1. Values of *p* determined by *t*-test. For all graphs, each dot represents samples from different animals.

We have previously demonstrated that enhancing AQP1 expression in PASMCs from control rats was sufficient to induce migration and proliferation (Lai et al., 2014; Yun et al., 2017). To test whether the same holds true for MVECs, we increased AQP1 levels in normal cells using adenoviral forced expression of wild-type AQP1. We previously demonstrated that HA-tagged AQP1 transduced by this viral construct is functionally intact and exhibits appropriate surface expression (Lai et al., 2014). Infecting MVECs with AdAQP1 resulted in a significant increase in HA-tagged (expressed) AQP1 protein levels (Figure 6A). Compared to cells infected with AdGFP, cells infected with AdAQP1 exhibited increased proliferation (Figure 6B) and migration (Figure 6C).

### Effect of Augmenting AQP1 on MVEC Barrier Function

**Figure 7:**
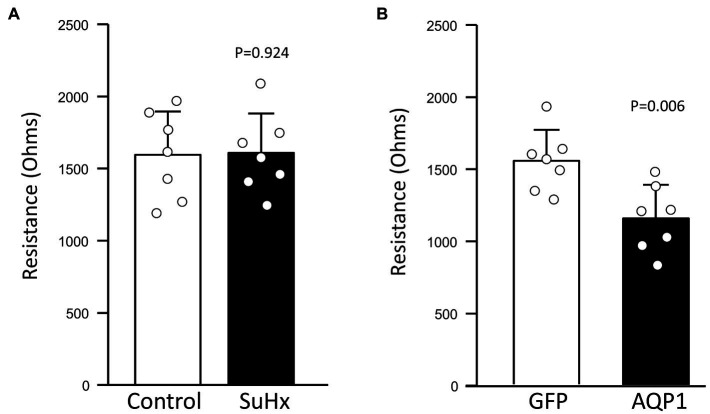
Effect of increasing AQP1 expression on endothelial barrier function. Bar graphs show mean ± SD values for baseline transendothelial resistance measured by electric cell-substrate impedance sensing (ECIS) in MVECs from **(A)** Control and SuHx rats and **(B)** from normal rats infected with adenovirus containing GFP (control) or AQP1. Values of *p* determined by *t*-test. For all graphs, each dot represents independent experiments with cells from different animals.

The main function of the endothelium is to provide a tight vascular barrier. Maintenance of appropriate barrier function and low permeability requires coordination between the cytoskeleton, cell-cell junction complexes, and cell attachments to the extracellular matrix and basement membrane. However, during periods of migration and proliferation, the stability of cell-cell junctions may be reduced to allow cell movement. To assess whether MVECs from SuHx rats exhibited reduced monolayer barrier function, we measured transendothelial resistance using ECIS. Comparison of baseline resistance between MVECs from Control and SuHx rats revealed no significant loss of barrier function (Figure 7A). We also tested the effect of augmenting AQP1 levels in control MVECs. Infection with AdGFP appeared to have no appreciable effect on baseline resistance, as levels measured in these cells (Figure 7B) were similar to those observed in uninfected control MVECs. However, cells infected with AdAQP1 consistently exhibited lower baseline resistance, suggesting that these cells had reduced ability to form a tight monolayer.

## Discussion

In this study, we explored the role of AQP1 in modulating pulmonary vascular cell migration and proliferation in a severe model of PH that captures many of the features of human PAH. We found that both PASMCs and MVECs from SuHx rats have enhanced expression of AQP1 that was associated with a more robust migratory and proliferative phenotype. We further showed that depleting AQP1 protein reduced migration and proliferation in these cells, whereas augmenting AQP1 expression in control cells was sufficient to mimic the SuHx phenotype. These results indicate an important role for AQP1 in controlling cell growth and movement during severe PH.

We previously demonstrated that exposure to chronic hypoxia upregulated AQP1 expression in PASMCs and contributed to hypoxia-induced migration and proliferation in this cell type (Leggett et al., 2012). This finding was confirmed in other labs (Schuoler et al., 2017; Liu et al., 2019), who also demonstrated that loss of AQP1 in the rodent lung, induced by antisense oligonucleotide (Schuoler et al., 2017) or transgenic approaches (Liu et al., 2019), reduced the development of hypoxia-induced PH. However, these studies have not evaluated the role of AQP1 in non-hypoxia-mediated PH. While the chronic hypoxia model captures features associated with Group 3 PH, it does not include the severe elevations in pulmonary arterial pressure, robust vascular remodeling, and development of vaso-occlusive lesions characteristic of human PAH. PAH is also not typically associated with hypoxia, except perhaps in late disease. Compared to chronic hypoxia and murine models, the SuHx rat model better recapitulates the features of human PAH; while hypoxia is necessary for model initiation, PH progresses after return to normoxic conditions, allowing separation of the direct effects of hypoxia from mechanisms likely to be involved in the maintenance and progression of PAH. In this model, despite a prolonged period of normoxia following the hypoxic exposure, we found that intralobar PAs had greater AQP1 protein expression than that measured in PAs from control rats (Figure 3) reminiscent of results published for the chronically hypoxic rat (Leggett et al., 2012).

The development of severe pulmonary vascular remodeling and occlusive lesions may be due to hyperproliferation/migration of PASMCs and/or MVECs, as positive staining for both SMC and EC markers in the cells forming occlusions has been reported (Taraseviciene-Stewart et al., 2001; Abe et al., 2010; Huetsch et al., 2016). Thus, we isolated both PASMCs and MVECs from control and SuHx rats to assess AQP1 expression. To our surprise, AQP1 protein expression in MVECs was remarkably low and often undetectable in control MVECs. Indeed, compared to PASMCs, we needed to load much larger amounts of protein per lane (25 μg vs. 10 μg) to be able to detect AQP1 protein in control MVECs. AQP1 protein expression has been demonstrated in pulmonary vascular ECs *in vivo* (King et al., 1996; Effros et al., 1997) or freshly isolated from the lung (Schnitzer and Oh, 1996), suggesting that in the native environment, these cells express some level of the protein constitutively. One possibility to explain this discrepancy is the potential for differential expression of AQP1 between MVECs and those from larger arteries, as has been demonstrated for other membrane channels (Stevens, 2011). Interestingly, we found that MVECs from control rats expressed ample mRNA transcript, with levels similar to those in PASMCs. Thus, the low levels of AQP1 protein measured in MVECs cannot be attributed to a lack of mRNA. Whether the discrepancy between AQP1 mRNA and protein levels measured in control MVECs reflects the loss of AQP1 protein expression due to culture conditions, removal of mechanical stimuli (i.e., shear stress), and/or differences in post-transcriptional synthesis or degradation pathways remains to be determined.

Despite differences in basal AQP1 levels in control cells, AQP1 protein levels were increased in both cell types from SuHx rats. The increase in AQP1 protein levels observed in MVECs from SuHx rats appears to be due, at least in part, to transcriptional upregulation, likely accounting for the increase in AQP1 mRNA observed in intact PAs. The mechanism by which AQP1 mRNA levels were augmented in MVECs from the SuHx rats is not presently known. The AQP1 promoter has been shown to contain a hypoxia response element and is directly regulated by HIFs (Abreu-Rodriguez et al., 2011; Tanaka et al., 2011), transcription factors that are stabilized during hypoxia and rapidly degraded with a return to normoxia (Semenza, 2012). Consistent with these previous findings, we found that exposure of MVECs from control rats to *in vitro* hypoxia induced a HIF-2-dependent increase in AQP1 protein expression. However, the durable increase in AQP1 mRNA levels in MVECs from SuHx rats, remaining evident 2 weeks after return to normoxia, suggests either a non-hypoxic stabilization of HIFs, perhaps secondary to mitochondrial dysfunction (Archer et al., 2008; Suresh et al., 2019), or could suggest that other factors aside from HIF activation maintain elevated AQP1 mRNA levels. Consistent with the former possibility, HIF levels are increased in PAECs from humans with PAH (Fijalkowska et al., 2010) and in the lungs of SuHx rats, although the cell type(s) involved was not explored (Dai et al., 2018). Whether the upregulation of AQP1 mRNA in MVECs from SuHx rats is due to HIF activation, or induction by another mechanism, also remains to be determined.

In contrast to the findings in MVECs, AQP1 mRNA levels were not statistically increased in PASMCs from SuHx rats despite augmented protein levels, reflecting apparent cell-type-specific regulation. This result is consistent with our previous findings showing hypoxia-induced increases in AQP1 protein were not accompanied by increased mRNA levels (Leggett et al., 2012). Others have shown a modest increase in AQP1 mRNA levels in PASMCs induced by *in vitro* exposure to hypoxia, although protein levels under these conditions were not examined (Abreu-Rodriguez et al., 2011). The lack of statistically significant induction of AQP1 mRNA levels in PASMCs from SuHx rats argues for the potential of post-transcriptional regulation, perhaps *via* enhanced translation or protein stabilization.

Our results indicate that elevated AQP1 protein levels are required for the hyperproliferative/hypermigatory phenotype observed in PASMCs and MVECs from the SuHx rat model. Moreover, using a viral construct to augment AQP1 levels in control MVECs demonstrated that elevations in AQP1 are sufficient to induce these changes in the absence of other stimuli, suggesting an increase in AQP1 levels could be an inciting event to modify cell function. Although we did not test the effect of loss of AQP1 protein *in vivo*, due to lack of appropriate inhibitors to reduce protein expression or availability of AQP1-null rats, in hypoxia-induced PH loss of AQP1 was shown to blunt the development of PH (Schuoler et al., 2017; Liu et al., 2019). Taken together with the findings from the SuHx rat, we speculate that upregulated AQP1 protein in PASMCs and MVECs during PAH may promote vascular remodeling through enhanced growth and migration of these cells.

It should be noted that the previous studies exploring the effect of deleting AQP1 on hypoxia-induced PH did not do so in a cell-type-specific manner, and various cells within the lung have been shown to express AQP1. For example, AQP1 expression has been reported in human and rat PASMCs (Leggett et al., 2012; Schuoler et al., 2017), macrophages (Rabolli et al., 2014; Tyteca et al., 2015), human activated T and B cells (Moon et al., 2004) and human and mouse type II pneumocytes (Effros et al., 1997; Galan-Cobo et al., 2018); however, staining for AQP1 was not observed in rat alveolar epithelium (Nielsen et al., 1997) or mouse type I cells (Effros et al., 1997). Thus, while the reduced development of hypoxia-induced PH in the previous studies could have resulted from the loss of AQP1 in PASMCs or MVECs, a role for AQP1 in other cell types cannot be ruled out at this point.

The main function of the endothelium is to create a tight barrier to prevent protein and fluid leakage into the tissue. AQP1 has been previously shown to play a role in regulating water permeability across the lung endothelium (Bai et al., 1999), although this was thought to be due to transcellular water transport. A hyperproliferative/hypermigratory phenotype, as observed in MVECs from SuHx rats or with AdAQP1 infection, might be expected to be associated with reduced cell-cell contact, or junction formation, to allow cell movement and thus increased paracellular permeability. To test this possibility, we measured basal transendothelial electrical resistance, a surrogate for paracellular permeability, and found no difference between MVEC isolated from Control and SuHx rats. This finding is consistent with a lack of reports of lung edema formation in SuHx rats or in PAH patients. On the other hand, increasing AQP1 in normal cells significantly reduced monolayer resistance, suggesting reduced cell-cell contact and increased paracellular permeability. This finding contrasts with recent work using human lung MVECs, where depletion of AQP1 was associated with increased monolayer permeability and reduced VE-cadherin expression (Gao et al., 2012). The reason for the discrepancy between our results and those of this previous study is unclear and suggests that further work is needed to fully delineate the role of AQP1 in maintaining endothelial barrier function. Similarly, the difference in our results between acute increases in AQP1 and those induced in cells from SuHx rats also warrants further study, perhaps pointing to compensatory mechanisms activated in PAH to prevent changes in paracellular permeability.

In summary, we found that AQP1 protein was upregulated in a model of PAH and contributed to enhanced migration and proliferation of pulmonary vascular cells. Similar to previous findings in hypoxia-induced PH (Schuoler et al., 2017; Liu et al., 2019), it is likely that AQP1 also mediates remodeling in the rat PAH model and points to a possible role for AQP1 in human PAH. Interestingly, the presence of a variant of AQP1 is associated with PAH in humans (Graf et al., 2018), although the effect of this variant on protein function/expression is currently not known. While these findings are intriguing, additional work will be needed to determine whether AQP1 could be a target for therapeutic intervention to treat human disease.

## Data Availability Statement

The raw data supporting the conclusions of this article will be made available by the authors, without undue reservation.

## Ethics Statement

The animal study was reviewed and approved by Johns Hopkins Animal Care and Use Committee.

## Author Contributions

LS and XY conceived of the experiments. LS, XY, JH, and KS designed the experiments. XY, JH, ZS, HJ, and NP performed the experiments. LS, XY, JH, KS, MD, and ZS analyzed the data and interpreted the results. LS and XY drafted the manuscript. LS, XY, JH, KS, HJ, NP, ZS, and MD revised the manuscript and approved the final version. All authors contributed to the article and approved the submitted version.

## Funding

This work was funded by grants from the National Institutes of Health (K08HL133475 to JH; K08HL132055 to KS; R01HL073859 and R01HL126514 to LS; and HL159906 to LS and MD) and the American Heart Association (18POST34030262 to XY).

## Conflict of Interest

JH is currently employed by Arrowhead Pharmaceutical. However, his participation in this study was conducted while he was employed by Johns Hopkins.

The remaining authors declare that the research was conducted in the absence of any commercial or financial relationships that could be construed as a potential conflict of interest.

## Publisher’s Note

All claims expressed in this article are solely those of the authors and do not necessarily represent those of their affiliated organizations, or those of the publisher, the editors and the reviewers. Any product that may be evaluated in this article, or claim that may be made by its manufacturer, is not guaranteed or endorsed by the publisher.
